# Studies on Cytotoxic Activity against HepG-2 Cells of Naphthoquinones from Green Walnut Husks of *Juglans mandshurica* Maxim

**DOI:** 10.3390/molecules200915572

**Published:** 2015-08-26

**Authors:** Yuanyuan Zhou, Bingyou Yang, Yanqiu Jiang, Zhaoxi Liu, Yuxin Liu, Xiaoli Wang, Haixue Kuang

**Affiliations:** 1 College of Pharmacy, Heilongjiang University of Chinese Medicine, Harbin 150040, China; E-Mails: zhouyuanyuan1998@163.com (Y.Z.); ybywater@163.com (B.Y.); jiangyanqiu219@163.com (Y.J.); liu_zhao_xi@163.com (Z.L.); ZYC19901014@126.com (Y.L.); 2 College of Adult Education, Heilongjiang University of Chinese Medicine, Harbin 150040, China; E-Mail: wangxiaolicando2000@126.com

**Keywords:** *Juglans mandshurica* Maxim, green walnut husks, naphthoquinones, cytotoxic activity, structure-activity relationships

## Abstract

Twenty-seven naphthoquinones and their derivatives, including four new naphthalenyl glucosides and twenty-three known compounds, were isolated from green walnut husks, which came from *Juglans mandshurica* Maxim. The structures of four new naphthalenyl glucosides were elucidated based on extensive spectroscopic analyses. All of these compounds were evaluated for their cytotoxic activities against the growth of human cancer cells lines HepG-2 by MTT [3-(4,5-dimethylthiazo l-2-yl)-2,5 diphenyl tetrazolium bromide] assay. The results were shown that most naphthoquinones in an aglycone form exhibited better cytotoxicity *in vitro* than naphthalenyl glucosides with IC_50_ values in the range of 7.33–88.23 μM. Meanwhile, preliminary structure-activity relationships for these compounds were discussed.

## 1. Introduction

With the increased use of natural product-based cancer chemotherapy, exploring the cytotoxic activity of phytochemicals for anticancer drug design has gained extensive attention worldwide [1]. *Juglans mandshurica* Maxim is a well-known member of the *Juglandaceae* family which is widely distributed throughout urban and rural areas in northeast China [2,3,4]. A few distrubute in Russia, Korea and Japan. It is one of the most important medical plants of which the green husks, leaf, root and bark all can be medically used [5,6,7,8]. Its green husks have been used as a folk medicine for treatment of gastric ulcers, uterine prolapse, leukopenia, diarrhea and dysentery for many years in China [9]. In recent years, many studies showed that green walnut husks have obvious advantages in tumor treatment like liver cancer [9,10,11].

Based on our interest in natural antitumor sources prompted us to continue investigating the phytochemicals and cytotoxicity of this plant [12,13,14,15,16]. A number of extracts and compounds obtained from medical materials have been identified as *in vitro* tumor inhibitors [7,9]. These beneficial effects have largely been ascribed to the presence of naphthoquinones. Naphthoquinones are the most important and widely distributed chemical class in the quinone family. Their derivatives have exhibited a variety of biological responses which include antiallergic, antibacterial, antifungal, anti-inflammatory, antithrombotic, antiplatelet, antiviral, apoptosis, lipoxygenase, radical scavenging, and anti-ringworm activities. Many studies have shown that naphthoquinones have biological activities specifically against pathogenic protozoa and cancer cells owing to their privileged structures in medicinal chemistry [17]. In this study, we obtained a series of naphthoquinone aglycones and glucosides, and then presented the isolation and structural elucidation of four new naphthalenyl glucosides (compounds **18**, **25**–**27**), together with 23 known compounds from green walnut husks of *J. mandshurica* Maxim. These included juglone (**1**), 5-methoxy-1,4-naphthoquinone (**2**), 5,8-dihydroxy-1,4-naphthoquinone (**3**), 2-hydroxy-1,4-naphthoquinone (**4**), 2,5-dihydroxy-1,4-naphthoquinone (**5**), 3,5-dihydroxy-1,4-naphthoquinone (**6**), 3-methoxy juglone (**7**), 2-methoxy juglone (**8**), 3-ethoxy juglone (**9**), 2-ethoxy juglone (**10**), Engelharquinone (**11**), (*S*)-regiolone (**12**), (4*S*)-4-hydroxy-α-tetralone (**13**), (4*S*)-5-hydroxy-4-methoxy-α-tetralone (**14**), 1,4,5-trihydroxynaphthalene-1,4-di-*O*-β-d-glucopyrano side (**15**), 1,4,5-trihydroxynaphthalene-1,5-di-*O*-β-d-glucopyranoside (**16**), 1,4,8-trihydroxynaphthalene-1-*O*-β-d-glucopyranoside (**17**), 1,4,8-trihydroxy-3-naphthalenecarboxylic acid -1-*O*-β-d-glucopyranoside ethyl ester (**18**), 1,4,8-trihydroxynaphthalene-1-*O*-β-d-[6′-*O*-(3′′,4′′,5′′-trihydroxybenzoyl)]glucopyranoside (**19**), (4*S*)-4-hydroxy-α-tetralone-4-*O*-β-d-glucopyranoside (**20**), (4*S*)-4,5-dihydroxy-α-tetralone 4-*O*-β-d-glucopyranoside (**21**), (4*S*)-4,6-dihydroxy-α-tetralone 4-*O*-β-d-glucopyranoside (**22**), (4*S*)-4,5,8-trihydroxy-α-tetralone 4-*O*-β-d-glucopyranoside (**23**), (4*S*)-4,5,8-trihydroxy-α-tetralone 5-*O*-β-d-[6′-*O*-(3′′,4′′,5′′-trihydroxybenzoyl)] glucopyranoside (**24**), (4*S*)-4-hydroxy-α-tetralone-4-*O*-β-d-(6′-*O*-4′′-hydroxylbenzoyl)glucopyranoside (**25**), (4*S*)-4,5-dihydroxy-α-tetralone-4-*O*-β-d-(6′-*O*-4′′-hydroxylbenzoyl)glucopyranoside (**26**), (4*S*)-4,5,8-thihydroxy-α-tetralone-5-*O*-β-d-(6′-*O*-4′′-hydroxylbenzoyl)glucopyranoside (**27**). In this study, we explored the antitumor structure-activity relationships using above compounds against the growth of liver cancer cell HepG-2 by MTT assays. The aim of this work was to define the key naphthoquinone structural elements that were required for cytotoxic activity through the determination of the ability of 27 naphthoquinones belonging to main structural subtypes such as naphthoquinone, tetralone, and naphthols.

## 2. Results and Discussion

### 2.1. Isolation and Characterization of Compounds **18**, **25**–**27**

**Figure 1:**
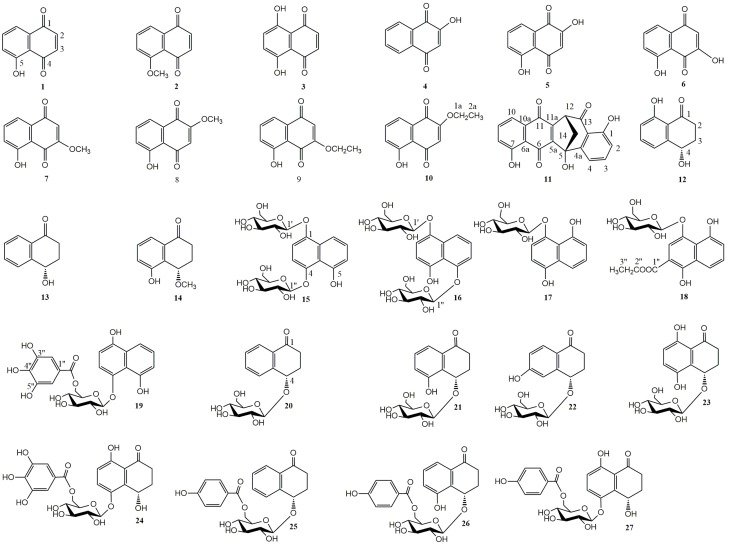
The chemical structures of compounds **1**–**27**.

**Figure 2:**
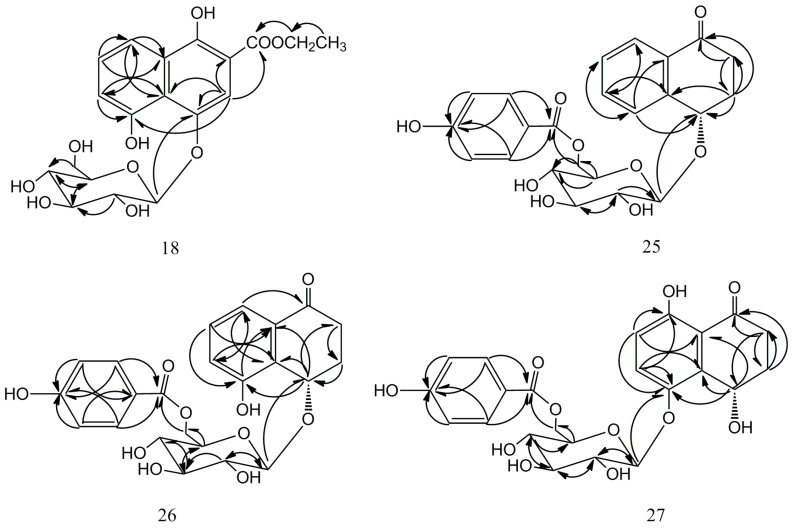
Key HMBC correlations of compounds **18**, **25**–**27**.

The compounds were isolated using silica gel columns and semi-preparative HPLC chromatography from 30% ethanol extract of fresh green husks of *Juglans mandshurica* Maxim. The structures of four new naphthalenyl glucosides were elucidated based on extensive mass and spectroscopic analyses including HR-ESI-MS, IR, ^1^H-NMR, ^13^C-NMR, DEPT, HSQC, HMBC, and CD. Their structures, ^1^H- and ^13^C-NMR data, and HMBC correlations are shown in Figure 1 and Figure 2 and Table 1.

Compound **18** was a red amorphous powder. The molecular formula C_19_H_22_O_10_ was determined from HR-ESI-MS and ^13^C-NMR data. There were two major differences between **18** and **25**–**27**: two methylene groups located at C-2 and C-3, respectively, at δ_C_ 33.0–35.0 and 30.0–31.5 in compounds **25**–**27** were replaced by methenyl groups at δ_C_ 109.9 and 105.8 in compound **18**, indicating no presence of a hydrogenated position. Furthermore, the independent existence of the glucopyranosyl moiety was not together with *p*-hydroxybenzoly on the basis of 1D-, 2D-NMR data. Noise-decoupled ^13^C-NMR and the distortionless enhancement by polarization transfer (DEPT) spectrum of **18** showed 19 carbon peaks, including one methyl, two methylenes, nine methynes, and seven quaternary carbons. There were 10 carbons due to the naphthalene ring, six carbons due to the glucose, and a carbonyl ketone at δ_C_ 171.8 correlated with one ethyl group, which was assigned to acetyl group. In the ^1^H-NMR spectrum, there were ABC-spin aromatic proton signals at δ_H_ 6.99 (dd, *J* = 1.0, 7.8 Hz, H-5), 7.40 (t, *J* = 7.8 Hz, H-6), and 7.86 (dd, *J* = 1.0, 7.8 Hz, H-7), which couple among themselves. Moreover, one isolated proton signal due to H-2 at δ_H_ 7.72 and one double-peak signal due to an anomeric proton at δ_H_ 4.99 were distinct. In the HMBC spectrum of **18** (Figure 2), the correlation peak between the anomeric proton and C-1 at δ_C_ 148.0 was observed. The results implied that the glucopyranosyl was linked to C-1 of the aglycone (Table 1, Figure 2). Thus, the structure of **18** was elucidated as 1,4,8-trihydroxy-3-naphthalenecarboxylic acid 1-*O*-β-d-glucopyranoside ethyl ester.

Compound **25** was obtained as a yellow amorphous powder and the molecular formula was assigned as C_23_H_24_O_9_ from its HR-ESI-MS and ^13^C-NMR data. ^1^H-NMR and ^13^C-NMR spectra revealed that **25** contained a typical β-d-glucopyranosyl (δ_H_ 4.42 (d, *J* = 7.6 Hz, H-1′); δ_C_ 103.7, 75.2, 78.1, 72.2, 75.5, 65.0), which was confirmed by acid hydrolysis and co-chromatography in comparison with an authentic sample. Moreover, the remaining 17 carbon signals, which respectively belong to the tetralone moiety and a *p*-hydroxybenzoly group, were attributable to two methylenes, nine methines, four olefinic quaternary carbons, and two quaternary carbonyl groups. To ascertain the structure of the aglycone and the glycosidic connection, a complete ^1^H- and ^13^C-NMR spectral assignment was carried out utilizing a combination of DEPT, HSQC, HMBC, and CD experiments. To be specific, the ^1^H-NMR spectrum of **25** showed two methylenes of tetralone at δ_H_ 2.87 (ddd, *J* = 4.5, 8.9, 17.5 Hz, H_ax_-2) and δ_H_ 2.41 (ddd, *J* = 4.5, 6.5, 17.5 Hz, H_eq_-2); 2.34 (dddd, *J* = 2.2, 4.5, 8.9, 13.4 Hz, H_ax_-3) and 2.28 (dddd, *J* = 3.8, 4.5, 6.5, 13.4 Hz, H_eq_-3), corresponding to C-atom signals at δ_C_ 35.5 and 31.5 in the HSQC spectrum. In the ^1^H-NMR spectrum, there was a set of correlation signals at δ_H_ 7.65 (br.d, *J* = 7.6 Hz, H-5), 7.52 (dt, *J* = 1.2, 7.6 Hz, H-6), 7.43 (dt, *J* = 1.2, 7.6 Hz, H-7), and 7.93 (dd, *J* = 1.2, 7.6 Hz, H-8) due to an *ortho*-disubstituted aromatic ring. All above data implied that **25** was an α-tetralone derivative. Hydrolysis of **25** yielded glucose, which was identified on a thin layer chromatography (TLC) plate by comparison with a reference sample. Moreover, a suggestive correlation was observed between the anomeric proton signal of glucose and a methane carbon signal at δ_C_ 75.9 (C-4) in the HMBC spectrum (Figure 2), indicating that the sugar moiety was linked at the C-4 position. The β-anomeric configuration for glucopyranose was determined from the *J*_H1,H2_ value (7.6 Hz). At the same time, it was also observed that the δ_H_ 4.66 (dd, *J* = 2.2, 11.8 Hz, H-6′a) and 4.47 (dd, *J* = 7.2 Hz, 11.8 Hz, H-6′b) had a linkage with formyl group. There were two sets of high peaks at δ_H_ 7.95 (d, *J* = 8.8 Hz, H-2′′, 6′′), 6.84 (d, *J* = 8.8 Hz, H-3′′, 5′′), and δ_C_132.9 (C-2′′, 6′′), 116.3 (C-3′′, 5′′) in the ^1^H- and ^13^C-NMR spectrum, indicating the presence of *p*-hydroxybenzoly. To determine the absolute configuration of the chiral center at the C-4 position, **25** was hydrolyzed to give the aglycone, which was identified to be *S* configuration by comparing its NMR data with those of the reference [18,19] and the circular dichrosim CD spectrum, where a negative Cotton effect at 236 nm was observed. On the basis of the above evidence, the structure of **25** was established as (4*S*)-4-hydroxy-α-tetralone 4-*O*-β-d-(6′-*O*-4′′-hydroxybenzoyl) glucopyranoside.

Compound **26**, a yellow amorphous powder, was assigned as C_23_H_24_O_10_ on the basis of its HR-ESI-MS and ^13^C-NMR data. The 1D- and 2D-NMR spectrographic data were similar as compound **25** except for the aryl ring moiety of the tetralone. The ^1^H-NMR spectrum of **26** showed a set of proton signals that was in accordance with the ABC-type aromatic proton signals, indicating the presence of a hydroxyl group at the C-5 position on the aromatic ring. The position of the hydroxyl group was also deduced to the C-5 position by observation of the correlations between δ_H_ 5.37 (H-4) and δ_C_ 157.0 (C-5) in the HMBC spectrum (Figure 2). The C-6, C-8, and C-10 located in the *ortho*- and *para*-position of C-5 were different from compound **25** due to the influence of the hydroxyl group. Moreover, the absolute configuration of **26** was determined as 4*S* from the CD spectrum of its aglycon [18], which had a negative Cotton effect. Thus, the structure of **26** was established as (4*S*)-4,5-dihydroxy-α-tetralone-4-*O*-β-d-(6′-*O*-4′′-hydroxybenzoyl) glucopyranoside.

Compound **27** was isolated as a yellow powder, which had the molecular formula C_23_H_24_O_11_, established in HR-ESI-MS. Hydrolysis of **27** was similar to **25** and **26**. Glucose was further confirmed by ^1^H-, ^13^C-NMR, and the DEPT spectrum (δ_H_ 4.81 (d, *J* = 7.5 Hz, H-1′); δ_C_ 104.4, 75.2, 78.0, 72.0, 75.8, 64.8). The correlation position between the aglycone and glucose was different from compounds **25** and **26**, which was deduced to transfer to δ_C_ 148.3 (C-5), implying the connection at the aryl ring of the tetralone by the HMBC spectrum. The relative configuration of the glucopyranose moiety was determined as β by the coupling constant (*J* = 7.5 Hz) of the anomeric proton. Furthermore, the ^1^H-NMR spectrum showed the AB-type aromatic proton signals at δ_H_ 7.40 (d, *J* = 9.1 Hz, H-6) and 6.67 (d, *J* = 9.1 Hz, H-7) in this aryl ring. It was also observed that a new quaternary carbon signal appeared at δ_C_ 159.3 due to the C-8 position in DEPT spectrum. It was also worth noting that the carbon signal at δ_C_ 116.2 (C-3′′, 5′′) not only had connections with H-3′′, 5′′ and H-2′′, 6′′, but also related with H-7 and H-4 (Figure 2) in HMBC. So we deduced that C-9 and C-3′′, 5′′ occurred in the same position. The absolute configuration of the chiral center at the C-4 position was deduced to be *S* by CD spectrum analysis of its aglycon [18]. Thus, the structure of **27** was determined to be (4*S*)-4,5,8-thihydroxy-α-tetralone 5-*O*-β-d-(6′-*O*-4′′-hydroxybenzoyl) glucopyranoside.

**Table 1:** ^1^H-(400 MHz) and ^13^C-(100 MHz) NMR data of **18**, **25**–**27** in CD_3_OD.

No.	18		25		26		27	
	δ_H_ (*J* in Hz)	δ_C_	δ_H_ (*J* in Hz)	δ_C_	δ_H_ (*J* in Hz)	δ_C_	δ_H_ (*J* in Hz)	δ_C_
**1**	—	148.0	—	200.0	—	200.9	—	206.4
**2**	7.72, s	109.9	H_ax_: 2.87, ddd (4.5, 8.9, 17.5)	35.5	H_ax_: 3.03, ddd (5.0, 13.4, 17.0)	33.9	H_ax_: 3.01, ddd (5.9,12.9, 17.6)	33.5
			H_eq_: 2.41, ddd (4.5, 6.5, 17.5)		H_eq_: 2.37, dt (3.6, 17.0)		H_eq_: 2.44, dt (3.6, 17.6)	
**3**	—	105.8	H_ax_: 2.34, dddd (2.2, 4.5, 8.9, 13.4)	31.5	H_ax_: 2.48, dddd (1.3, 3.2, 4.7, 12.6)	30.0	2.16, m	30.3
			H_eq_: 2.28, dddd (3.8, 4.5, 6.5, 13.4)		H_eq_: 2.10, tt (4.2, 12.6)			
**4**	—	155.1	4.97, dd (3.6, 6.5)	75.9	5.37, t (2.9)	69.9	5.32, t (3.1)	61.3
**5**	6.99, dd (1.0, 7.8)	116.0	7.65, brd (7.6)	130.0	—	157.0	—	148.3
**6**	7.40, t (7.8)	128.6	7.52, dt (1.2, 7.6)	134.8	7.08, dd (0.8, 8.0)	122.3	7.40, d (9.1)	128.9
**7**	7.86, dd (1.0, 7.8)	116.1	7.43, dt (1.2, 7.6)	129.4	7.27, t (8.0)	130.6	6.67, d (9.1)	118.9
**8**	—	158.0	7.93, dd (1.2, 7.6)	127.9	7.46, dd (0.8, 8.0)	118.9	—	159.3
**9**	—	120.2	—	132.9	—	134.4	—	116.2
**10**	—	131.1	—	143.9	—	129.6	—	135.5
**1′**	4.99, d (7.6)	105.5	4.42, d (7.6)	103.7	4.60, d (7.8)	103.8	4.81, d (7.5)	104.4
**2′**	3.50, m	78.9	3.34, m	75.2	3.2, m	75.3	3.53, dd (8.8, 16.5)	75.2
**3′**	3.53, m	75.0	3.36, dd (2.5, 7.0)	78.1	3.38, m	78.0	3.49, t (8.6)	78.0
**4′**	3.43, m	71.3	3.36, dd (2.5, 7.0)	72.2	3.38, m	72.1	3.43, dd (10.6, 16.2)	72.0
**5′**	3.48, m	78.2	3.62, m	75.5	3.65, m	75.7	3.68, dt (2.2, 8.4)	75.8
**6′a**	3.96, dd (2.1, 12.0)	62.5	4.66, dd (2.2, 11.8)	65.0	4.64, dd (2.2, 11.8)	65.0	4.61, dd (2.2, 11.8)	64.8
**6′b**	3.77, dd (5.5, 12.0)		4.47, dd (7.2, 11.8)		4.43, dd (6.8, 11.8)		4.40, dd (7.4, 11.8)	
**1′′**	—	171.8	—	122.2	—	121.9	—	122.1
**2′′**	4.44, dq (3.6, 17.7)	62.8	7.95, d (8.8)	132.9	7.95, dt (2.6, 8.8)	133.0	7.82, dt (2.7, 8.8)	132.9
**3′′**	1.43, t (7.2)	14.5	6.84, d (8.8)	116.3	6.83, dt (2.6, 8.8)	116.2	6.81, dt (2.7, 8.8)	116.2
**4′′**	—	—	—	163.7	—	163.6	—	163.7
**5′′**	—	—	6.84, d (8.8)	116.3	6.83, dt (2.6, 8.8)	116.2	6.81, dt (2.7, 8.8)	116.2
**6′′**	—	—	7.95, d (8.8)	132.9	7.95, dt (2.6, 8.8)	133.0	7.82, dt (2.7, 8.8)	132.9
**7′′**	—	—	—	167.9	—	168.1	—	167.8

### 2.2. Cytotoxic Activity

It was reported that green husks of *Juglans mandshurica* Maxim had an obvious effect on liver cancer. HepG-2 is a kind of human liver cancer cells which are often applied to evaluate cytotoxic activity *in vitro* [20,21].Therefore, we tested the cytotoxicity of compounds **1**–**27** against HepG-2 by the MTT method and compared with references for some compounds [22,23,24].

The results were shown that most naphthoquinones in an aglycone form exhibited better cytotoxicity *in vitro* than naphthalenyl glucosides with IC_50_ values in the range of 7.33–88.23 μM. None of them had better IC_50_ values than cisplatin itself, but some naphthoquinone aglycones including juglone (**1**) and 3,5-dihydroxy-1,4-naphthoquinone (**6**) had obvious inhibition effects similar with cisplatin. The IC_50_ value of juglone was 8.14 ± 1.95, and that of 3,5-dihydroxy-1,4-naphthoquinone was 7.33 ± 0.52 at 24 h of MTT assay, respectively (Table 2). Furthermore, these naphthoquinone aglycones with the structural features of 2,3-unsaturated moieties showed better and stronger cytotoxicity effects compared to other tetralones with a partial saturated aryl ring.

**Table 2:** Cytotoxicities of compound **1**–**27** from *J. mandshurica* Maxim on HepG-2 cells lines.

Compd.	Structural Features	IC_50_ (μM) ^a^	SD ^b^	Compd.	Structural Features	IC_50_ (μM) ^a^	SD ^b^
**1**	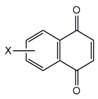	8.14	1.95	**15**	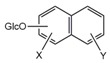	NA	3.21
**2**		68.72	1.50	**16**		NA	-
**3**		16.11	3.54	**17**		83.32	4.54
**4**		18.83	2.98	**18**		NA	-
**5**		15.37	1.63	**19**		78.61	2.38
**6**	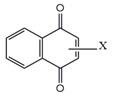	7.33	0.52	**20**	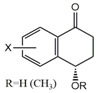	NA	-
**7**		43.54	0.15	**21**		NA	-
**8**		22.38	0.66	**22**		NA	-
**9**		30.42	2.48	**23**		NA	-
**10**		32.51	0.46	**24**		NA	-
**11**		34.80	0.33	**25**		NA	-
**12**	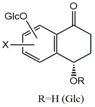	56.87	4.27	**26**		NA	-
**13**		67.95	3.22	**27**		NA	-
**14**		88.23	1.90	PC ^c^	metal complex	4.51	0.38

^a^ IC_50_, concentration required for inhibiting growth of HepG-2 by 50% (in μM). These results are average results of three experiments; ^b^ SD, standard deviation; ^c^ PC, positive control (cisplatin); NA = not active.

Above results were merely obtained from the distinction of the mother nucleus structure. The different nature of the substituent in the naphthoquinone also seemed to influence the cytotoxicity activity. One or two phenolic hydroxyl groups without other substituents, which were introduced to a set of analogues (compounds **1**, **3**, **4**, **5** and **6**), were responsible for the lower IC_50_ value and better inhibition effect. However, it was worth noting that the position and number of the hydroxyl group had a limited or negligible effect on HepG-2 inhibitory activities. For example, the IC_50_ value of compound **1** with one hydroxyl group was similar with compound **6** with two hydroxyl groups; the IC_50_ value of compound **3** which was substituted at the 5, 8-position was similar to compound **5** which was substituted at the 2, 5-position. In addition, the introduction of the methoxy or ethyoxyl group to some naphthoquinones with 2, 3-unsaturated moieties resulted in a slight decrease in the inhibition effect, including compounds **7**, **8**, **9** and **10**. Among of them, compound **2** had the worst effect on the inhibition of HepG-2 cells.

A majority of naphthoquinone glycosides exhibited no activity against HepG-2 cells. These results were in accordance with previous reports [24] that the most active compounds were those without the linkage of saccharide. Some glycosides like compound **17** and **19**, of which aglycone was the integrated conjugation structure assigned to the naphthols, possessed slight cytotoxicity *in vitro* with IC_50_ values of 83.3 ± 4.54 and 78.61 ± 2.38, respectively. However, some naphthols substituted with more than one saccharide or other groups, except for phenolic hydroxyl groups, had no cytotoxic activity against HepG-2 cells. The results indicated that there were differences in cytotoxic activity between these naphthoquinone glycosides according to the way of substitution and the type of aglycone.

## 3. Experimental Section

### 3.1. General Information

High resolution-electron spray ionization (HR-ESI) mass spectra were obtained on a micromass LCT spectrometer. ^1^H-, ^13^C-NMR, DEPT, HSQC, HMBC were obtained on Bruker DPX 400 NMR instrument (Bruker, Rheinstetten, Germany). Chemical shifts (δ) are expressed in parts per million (ppm) using tetramethyl-silane (TMS) as an internal standard. Spin multiplicities are given as s (singlet), d (doublet), t (triplet), dd (double doublet), and m (multiplet). The UV spectra were recorded on a Thermo Scientific Evolution 300 UV-visible spectrophotometer (Thermo Fisher Scientific, Waltham, MA, USA). Optical rotations were recorded using an Anton Paar-MCP 600 polarimeter r (Anton Paar, Graz, Austria). GC was run on Agilent 7890A Gas Chromatograph System (Agilent Technologies, Santa Clara, CA, USA). CD spectra were obtained on a Bio-Logic MOS-450 CD spectrometer. The IR spectra were obtained on a Shimadzu FTIR-8400S spectrometer (Shimadzu, Kyoto, Japan). Melting points are uncorrected and were obtained on a Hoover capillary melting point. HPLC chromatograms were obtained with an Agilent Technologies 1260 infinity HPLC system (Agilent Technologies, Germany) and semi-preparative HPLC (Waters, 515-2414, Milford, MA, USA) was performed using a Hypersil-ODS II column (300 mm × 20 mm i.d., 10 μm, Ylite, Dalian, China). De-ionized water was prepared with a Milli-Q system (Milford, MA, USA). HepG-2 cell line obtained from Institute of Biochemistry and Cell Biology (Shanghai, China) were grown in Dulbecco’s Modified Eagle’s Medium (DMEM) (Hyclone, NRH0020), supplemented with 5% fetal bovine serum and 1% antibiotic mixture comprising penicillin-streptomycin, in a humidified atmosphere at 37 °C with 5% CO_2_. A multiscan microplate reader (Thermo Labsystems, Helsinki, Finland) was used for the MTT assays. The solvents used for open column isolation, such as ethyl acetate, methanol, acetonitrile, and chloroform were purchased from Merck (Darmstadt, Germany). MTT and Dulbecco’s Modified Dagle’s Medium (DMEM) were purchased from Sigma Chemical Co. (St. Louis, MO, USA).

### 3.2. Plant Material

The green husks of *J. mandshurica* were collected in late July from the Changbai Mountains (Jilin, China), and identified by the professor Zhen-Yue Wang. The dried samples were grounded into fine powder (60 mesh), and dried thoroughly in an oven at 40 °C for 3 days.

### 3.3. Extraction and Isolation

The air-dried parts of materials (10.0 kg) were powdered and soaked in 80 L of CHCl_3_ for 7 days. The extraction was repeated three times and then concentrated under reduced pressure to afford the CHCl_3_ extract (350 g). CHCl_3_ extract was subjected to silica gel (200–300 mesh) column chromatography (CC), eluted with PE:EtOAc (40:1→1:1, *v*/*v*), to create twelve fractions (Fr1-Fr12). Fraction 3 (15.50 g) was subjected to silica gel (200–300 mesh) CC, eluted with PE:EtOAc (40:1→1:1, *v*/*v*), to give fractions 3a–3c. Compounds **1** (245.4 mg), **3** (12.1 mg), **4** (33.8 mg), and **8** (11.8 mg), were isolated from fraction 3a by repeated column chromatography over silica gel, eluted with PE:EtOAc (40:1→10:1, *v*/*v*). Fraction 6 (12.80 g) was subjected to silica gel (200–300 mesh) CC, eluted with PE:EtOAc (20:1→5:1, *v*/*v*) to obtain compounds **5** (15.5 mg), **7** (22.0 mg), **9** (25.3 mg), **10** (15.2 mg), **12** (75.0 mg), and **13** (23.4 mg). Fraction 8 (8.92 g) was subjected to silica gel (200–300 mesh) CC, eluted with PE:EtOAc (5:1→1:1, *v*/*v*) to obtain **2** (5.8 mg), **6** (7.7 mg), **11** (6.8 mg), and **14** (12.8 mg).

The residue of materials were reflux extracted three times with 60 L EtOH (95% *v*/*v*), then concentrated under reduced pressure to afford the EtOH extract (750 g). The EtOH extract was subjected to Macroporous Resin AB-8 CC, sequentially eluted with H_2_O, 30% EtOH, and 95% EtOH. Compounds **15**–**27** were isolated from 30% EtOH fraction. Next, the isolation procedure of these compounds was explained. The 30% EtOH elution fraction was evaporated and concentrated to yield a crude residue (98 g). The residue was further purified by octadecyl silane (ODS) CC with MeOH/H_2_O (2:8→1:0) to give eleven fractions (Fr1-Fr11). Fraction 2 (6.50 g) was fractionated by ODS CC with MeOH/H_2_O (2:8→1:0) to afford a number of subfractions: 2a, 2b, 2c. Subfraction 2c (1.0 g) was subjected to semi-preparative HPLC chromatography (MeOH/H_2_O 35:65, *v*/*v*, flow rate 3 mL/min) to yield compounds **15** (5.3 mg, t_R_ = 21 min), **16** (6.1 mg, t_R_ = 23 min), and then subjected to semi-preparative HPLC chromatography (MeOH/H_2_O 45:55, *v*/*v*, flow rate 3 mL/min) to yield compound **19** (3.2 mg, t_R_ = 22 min). Fraction 5 (9.40 g) was fractionated twice by ODS CC with MeOH/H_2_O (2:8→1:0) to afford a number of subfractions: 5a, 5b, 5c, 5d and 5e. Subfraction 5b (0.84 g) was purified by semi-preparative HPLC chromatography (MeOH/H_2_O 45:55, *v*/*v*, flow rate 3 mL/min) to yield compound **24** (4.7 mg, t_R_ = 32 min) and purified by semi-preparative HPLC chromatography (MeOH/H_2_O 55:45, *v*/*v*, flow rate 3 mL/min) to obtain compound **17** (4.3 mg, t_R_ = 25 min). Similarly, Subfraction 5c was purified by semi-preparative HPLC chromatography (MeOH/H_2_O 70:30, *v*/*v*, flow rate 3 mL/min, t_R_ = 25 min) to yield compounds **18** (4.5 mg, t_R_ = 13 min), **26** (2.8 mg, t_R_ = 38 min), **25** (3.1 mg, t_R_ = 40 min), and **27** (5.9 mg, t_R_ = 42 min). Fraction 8 (5.70 g) was subjected to silica gel (200–300 mesh) CC, eluted with CH_2_Cl_2_:MeOH (5:1→0:1, *v*/*v*) to afford compounds **20** (7.5 mg), **21** (5.3 mg), **22** (4.6 mg), **23** (4.4 mg).

### 3.4. Spectral Data

*Juglone* (**1**). Orange needle crystal, ^1^H-NMR (CDCl_3_, 400 MHz) δ (ppm): 6.99 (2H, d, *J* = 12.2 Hz, H-2, 3), 7.27 (1H, dd, *J* = 1.9, 7.7 Hz, H-6), 7.65 (1H, t, *J* = 7.6 Hz, H-7), 7.63 (1H, dd, *J* = 7.6, 7.7 Hz, H-8), 11.90 (1H, s, 5-OH). ^13^C-NMR (CDCl_3_, 100 MHz) δ (ppm): 190.3 (C-1), 138.7 (C-2), 139.6 (C-3), 184.3 (C-4), 161.5 (C-5), 124.5 (C-6), 136.6 (C-7), 119.2 (C-8), 115.1 (C-9), 131.8 (C-10).

*5-Methoxy-1,4-naphthoquinone* (**2**). Light yellow powder, ^1^H-NMR (CDCl_3_, 400 MHz) δ: 7.58 (1H, d, *J* = 7.6 Hz, H-2), 7.48 (1H, d, *J* = 7.6 Hz, H-3), 6.73 (1H, br.d, *J* = 7.7 Hz, H-6), 7.36 (1H, t, *J* = 7.7 Hz, H-7), 7.42 (1H, br.d, *J* = 7.7 Hz, H-8), 3.83 (3H, s, 5-OCH_3_), 11.82 (1H, s, 5-OH). ^13^C-NMR (CDCl_3_, 100 MHz) δ (ppm): 190.4 (C-1), 138.3 (C-2), 139.3 (C-3), 186.3 (C-4), 161.2 (C-5), 119.2 (C-6), 124.3 (C-7), 136.1 (C-8), 131.4 (C-9), 114.0 (C-10), 55.4 (5-OCH_3_).

*5,8-Dihydroxy-1,4-naphthoquinone* (**3**). Light yellow powder, ^1^H-NMR (CDCl_3_, 400 MHz) δ (ppm): 7.13 (4H, s, H-2, 3, 6, 7). ^13^ C-NMR (CDCl_3_, 100 MHz) δ (ppm): 173.1 (C-1, 4, 5, 8), 134. 4 (C-2, 3, 6, 7), 112. 0 (C-9, 10).

*2-Hydroxy-1, 4-naphthoquinone* (**4**)*.* Light yellow powder, ^1^H-NMR (CDCl_3_, 400 MHz) δ (ppm): 7.38 (1H, s, H-3), 8.13 (2H, d, *J* = 7.3 Hz, H-5, 8), 7.82 (1H, m, H-6), 7.74 (1H, m, H-7). ^13^C-NMR (CDCl_3_, 100 MHz) δ (ppm): 182.2 (C-1), 156.5 (C-2), 110.7 (C-3), 185.0 (C-4), 135.3 (C-5), 126.8 (C-6), 126.7 (C-7), 133.4 (C-8), 130.0 (C-9), 129.5 (C-10).

*2,5-Dihydroxy-1, 4-naphthoquinone* (**5**). Light yellow powder, ^1^H-NMR (CDCl_3,_ 400 MHz) δ (ppm): 6.12 (1H, s, H-3), 7.23 (1H, d, *J* = 8.2 Hz, H-6), 7.57 (1H, m, H-7), 7.40 (1H, d, *J* = 7.6 Hz, H-8), 12.10 (1H, s, 5-OH). ^13^C-NMR (CDCl_3_, 100 MHz) δ (ppm): 180.3 (C-1), 160.2 (C-2), 110.4 (C-3), 191.7 (C-4), 159.3 (C-5), 124.2 (C-6), 135.3 (C-7), 118.2 (C-8), 130.3 (C-9), 113.7 (C-10).

*3,5-Dihydroxy-1, 4-naphthoquinone* (**6**). Light yellow powder, ^1^H-NMR (CDCl_3_, 400 MHz) δ (ppm): 6.14 (1H, s, H-2), 7.26 (1H, d, *J* = 7.8 Hz, H-6), 7.66 (1H, t, *J* = 7.8 Hz, H-7), 7. 43 (1H, d, *J* = 7.8 Hz, H-8), 12.02 (1H, s, 5-OH). ^13^C-NMR (CDCl_3_, 100 MHz) δ (ppm): 183.0 (C-1), 160.0 (C-2), 111.4 (C-3), 185.3 (C-4), 160.3 (C-5), 122.2 (C-6), 136.0 (C-7), 117.0 (C-8), 132.5 (C-9), 114.2 (C-10).

*3-Methoxy juglone* (**7**). Orange powder, ^1^H-NMR (CDCl_3_, 400 MHz) δ (ppm): 6.12 (1H, s, H-2), 7.27 (1H, dd, *J* = 7.5, 1.2 Hz, H-6), 7.62 (1H, t, *J* = 7.5 Hz, H-7), 7.67 (1H, d, *J* = 7.5, 1.2 Hz, H-8), 3.90 (3H, s, OCH_3_), 11.75 (1H, s, OH-5). ^13^C-NMR (CDCl_3_, 100 MHz) δ (ppm): 184.9 (C-1), 110.5 (C-2), 160.6 (C-3), 183.9 (C-4), 162.0 (C-5), 123.9 (C-6), 138.2 (C-7), 118.9 (C-8), 132.1 (C-9), 114.3 (C-10), 56.6 (3-OCH_3_).

*2-Methoxy juglone* (**8**). Orange-red needle crystal, ^1^H-NMR (CDCl_3_, 400 MHz) δ (ppm): 6.11 (1H, s, H-3), 7.28 (1H, dd, *J* = 8, 1.2 Hz, H-6), 7.58 (1H, t, *J* = 8 Hz, H-7), 7.67 (1H, dd, *J* = 8, 1.2 Hz, H-8), 3.93 (3H, s, 2-OCH3), 12.22 (1H, s, 5-OH). ^13^C-NMR (CDCl_3_, 100 MHz) δ (ppm): 179.3 (C-1), 161.1 (C-2), 109.5 (C-3), 190.8 (C-4), 161.1 (C-5), 125.2 (C-6), 135.4 (C-7), 119.5 (C-8), 131.1 (C-9), 114.2 (C-10), 56.6 (2-OCH_3_).

*3-Ethoxy juglone* (**9**). Orange powder, ^1^H-NMR (CDCl_3_, 400 MHz) δ (ppm): 6.13 (H, s, H-2), 7.24 (1H, dd, *J* = 7.5, 1.2 Hz, H-6), 7.62 (1H, t, *J* = 7.5 Hz, H-7), 7.62 (1H, dd, *J* = 7.5, 1.2 Hz, H-8), 4.11 (3H, q, *J* = 6.9 Hz, H-1a), 1.60 (2H, t, *J* = 6.9 Hz, H-2a), 11.78 (1H, s, OH-5). ^13^C-NMR (CDCl_3_, 100 MHz) δ (ppm): 185.1 (C-1), 110.8 (C-2), 159.3 (C-3), 184.1 (C-4), 161.9 (C-5), 123.8 (C-6), 118.8 (C-7), 137.1 (C-8), 132.0 (C-9), 114.3 (C-10), 65.6 (C-1a), 13.9 (C-2a).

*2-Ethoxy juglone* (**10**). Light yellow flaky crystal, ^1^H-NMR (CDCl_3_, 400 MHz) δ (ppm): 6.08 (1H, s, H-3), 7.67 (1H, dd, *J* = 7.5, 1.2 Hz, H-6), 7.59 (1H, t, *J* = 7.5 Hz, H-7), 7.26 (1H, dd, *J* = 7.5, 1.2 Hz, H-8), 4.12 (2H, q, *J* = 7.2 Hz, -OCH_2_-), 1.53 (3H, t, *J* = 7.2 Hz, -CH_3_), ^13^C-NMR (CDCl_3_, 100 MHz) δ (ppm): 190.0 (C-1), 160.1 (C-2), 109.3 (C-3), 179.2 (C-4), 161.0 (C-5), 114.6 (C-6), 135.3 (C-7), 125.0 (C-8), 131.1 (C-9), 119.0 (C-10), 65.5 (C-1′), 13.8 (C-2′).

*Engelharquinone* (**11**). Yellow needle crystal, ^1^H-NMR (CDCl_3_, 400 MHz) δ (ppm): 6.92 (1H, d, *J* = 8.4 Hz, H-2), 7.48 (1H, dd, *J* = 7.4, 8.4 Hz, H-3), 7.14 (1H, d, *J* = 7.4 Hz, H-4), 7.25 (1H, dd, *J* = 7.6, 1.9 Hz, H-8), 7.59 (1H, dd, *J* = 7.6, 7.4 Hz, H-9), 7.65 (1H, dd, *J* = 7.6, 1.9 Hz, H-10), 4.23 (1H, d, *J* = 3.1 Hz, H-12), 3.08 (1H, dd, *J* = 3.8, 10.6 Hz, H-14a), 3.04 (1H, brd, *J* = 3.8, 10.6 Hz, H-14b), 11.50 (1H, s, 1-OH), 4.80 (1H, s, 5-OH), 11.52 (1H, s, 7-OH). ^13^C-NMR (CDCl_3_, 100 MHz) δ (ppm): 198.1 (C-13), 188.8 (C-6), 180.5 (C-11), 163.5 (C-1), 162.1 (C-7), 154.4 (C-5a), 148.7 (C-11a), 146.3 (C-4a), 137.3 (C-9), 136.8 (C-3), 132.6 (C-11a), 125.2 (C-8), 120.4 (C-10), 119.4 (C-2), 115.3 (C-6a), 110.5 (C-13a), 82.0 (C-5), 54.1 (C-14), 52.6 (C-12).

*(S)-Regiolone* (**12**). White powder, ^1^H-NMR (DMSO-*d*_6_, 400 MHz) δ (ppm): 2.74 (2H, m, H-2), 2.20 (1H, m, H-3a), 2.00 (1H, m, H-3b), 4.76 (1H, m, H-4), 7.06 (1H, d, *J* = 8.0 Hz, H-5), 7.53 (1H, t, *J* = 8.0, 8.2 Hz, H-6), 6.83 (1H, d, *J* = 8.2 Hz, H-7), 5.60 (H, s, OH-4), 12.42 (1H, s, OH-8). ^13^C-NMR (DMSO-*d*_6_, 100 MHz) δ (ppm): 205.5 (C-1), 35.5 (C-2), 31.8 (C-3), 66.6 (C-4), 117.8 (C-5), 137.0 (C-6), 116.1 (C-7), 162.0 (C-8), 149.4 (C-9), 115.3 (C-10).

*(4S)-4-Hydroxy-α-tetralone* (**13**). Claybank oil substance, ^1^H-NMR (CDCl_3_, 400 MHz) δ (ppm): 2.53 (1H, ddd, *J* = 17.8, 9.6, 4.8 Hz, H-2a), 2.86 (1H, ddd, *J* = 17.8, 7.5, 4.6 Hz, H-2), 2.15 (1H, m, H-3a), 2.37 (1H, m, H-3b), 4.95 (1H, dd, *J* = 8.1, 3.9 Hz, H-4), 7.52 (1H, m, H-5, 6), 7.38 (1H, m, H-7), 7.98 (1H, brd, *J* = 7.8 Hz, H-8). ^13^C-NMR (CDCl_3_, 100 MHz) δ (ppm): 197.8 (C-1), 35.2 (C-2), 32.3 (C-3), 67.7 (C-4), 126.9 (C-5), 134.2 (C-6), 127.3 (C-7), 128.5 (C-8), 130.8 (C-9), 145.5 (C-10).

*(4S)-5-Hydroxy-4-methoxy-α-tetralone* (**14**). White amorphous powder, ^1^H-NMR (CDCl_3_, 400 MHz) δ (ppm): 2.63 (1H, m, H-2), 2.87 (1H, m, H-2), 2.11 (1H, m, 3a), 2.24 (1H, m, H-3b), 4.98 (1H, m, H-4), 7.07 (1H, dd, *J* = 8.0, 1.0 Hz, H-6), 7.58 (1H, dd, *J* = 8.0, 1.0 Hz, H-7), 7.33 (1H, t, *J* = 8.0 Hz, H-8), 8.55 (1H, s, 5-OH), 3.57 (3H, s, 4-OCH_3_). ^13^C-NMR (CDCl_3_, 100 MHz) δ (ppm): 196.2 (C-1), 35.6 (C-2), 27.3 (C-3), 80.2 (C-4), 156.3 (C-5), 122.32 (C-6), 129.6 (C-7), 119.3 (C-8), 132.5 (C-9), 126.7 (C-10), 55.3 (C-11).

*1,4,5-Trihydroxynaphthalene-1,4-di-O-β-d-glucopyranoside* (**15**). Yellow amorphous powder, ^1^H-NMR (CD_3_OD, 400 MHz) δ (ppm): 7.16 (1H, d, *J* = 8.8 Hz, H-2), 7.30 (1H, d, *J* = 8.8 Hz, H-3), 6.85 (1H, dd, *J* = 7.6, 1.2 Hz, H-6), 7.34 (1H, dd, *J* = 8.4, 7.6 Hz, H-7), 7.89 (1H, dd, *J* = 8.4, 1.2 Hz, H-8), 5.04 (1H, d, *J* = 7.7 Hz, H-1′), 3.63 (1H, dd, *J* = 9.0, 7.7 Hz, H-2′), 3.53 (1H, dd, *J* = 9.0, 8.8 Hz, H-3′), 3.45 (1H, m, H-4′), 3.44 (1H, m, H-5′), 3.73 (1H, dd, *J* = 11.8, 5.8 Hz, H-6′a), 3.89 (1H, br.d, *J* = 11.8 Hz, H-6′b), 5.06 (1H, d, *J* = 7.8 Hz, H-1″), 3.58 (1H, dd, *J* = 9.0, 7.8 Hz, H-2″), 3.50 (1H, dd, *J* = 9.0, 8.5 Hz, H-3″), 3.43 (1H, m, H-4″), 3.46 (1H, m, H-5″), 3.74 (1H, dd, *J* = 12.4, 5.2 Hz, H-6″a), 3.94 (1H, dd, *J* = 12.4, 1.8 Hz, H-6″b). ^13^C-NMR (CDCl_3_, 100 MHz) δ (ppm): 150.9 (C-1), 111.3 (C-2), 112.2 (C-3), 151.0 (C-4), 155.1 (C-5), 112.5 (C-6), 128.3 (C-7), 114.8 (C-8), 130.4 (C-9), 117.5 (C-10), 103.0 (C-1′), 75.0 (C-2′), 78.5 (C-3′), 71.4 (C-4′), 78.5 (C-5′), 62.4 (C-6′), 105.4 (C-1″), 75.0 (C-2″), 78.4 (C-3″), 71.4 (C-4″), 78.9 (C-5″), 62.5 (C-6″).

*1,4,5-Trihydroxynaphthalene-1,5-di-O-β-d-glucopyranoside* (**16**). Yellow amorphous powder, ^1^H-NMR (CD_3_OD, 400 MHz) δ (ppm): 7.20 (1H, d, *J* = 8.2 Hz, H-2), 6.72 (1H, d, *J* = 8.2 Hz, H-3), 7.41 (1H, dd, *J* = 7.8, 1.6 Hz, H-6), 7.36 (1H, dd, *J* = 8.0, 7.8 Hz, H-7), 8.13 (1H, dd, *J* = 8.0, 1.6 Hz, H-8), 4.92 (1H, d, *J* = 7.8 Hz, H-1′), 3.60 (1H, dd, *J* = 8.7, 7.9 Hz, H-2′), 3.49 (1H, m, H-3′), 3.45 (1H, m, H-4′), 3.42 (1H, m, H-5′), 3.70 (1H, dd, *J* = 12.2, 5.1 Hz, H-6′a), 3.88 (1H, dd, *J* = 12.2, 2.0 Hz, H-6′b), 5.11 (1H, d, *J* = 7.9 Hz, H-1″), 3.58 (1H, dd, *J* = 9.2, 7.9 Hz, 2″), 3.50 (1H, m, H-3″), 3.43 (1H, m H-4″), 3.52 (1H, m, H-5″), 3.76 (1H, dd, *J* = 12.2, 5.8 Hz, H-6″a), 3.97 (1H, dd, *J* = 12.2, 2.1 Hz, H-6″b). ^13^C-NMR (CDCl_3_, 100 MHz) δ (ppm): 147.4 (C-1), 114.0 (C-2), 110.9 (C-3), 150.6 (C-4), 155.8 (C-5), 112.7 (C-6), 126.6 (C-7), 119.4 (C-8), 130.2 (C-9), 117.3 (C-10), 104.0 (C-1′), 75.2 (C-2′), 78.2 (C-3′), 71.5 (C-4′), 78.3 (C-5′), 62.4 (C-6′), 104.6 (C-1″), 75.2 (C-2″), 78.3 (C-3″), 71.5 (C-4″), 78.8 (C-5″), 62.4 (C-6″).

*1,4,8-Trihydroxynaphthalene-1-O-β-d-glucopyranoside* (**17**). Yellow amorphous powder, ^1^H-NMR (CD_3_OD, 400 MHz) δ (ppm): 6.70 (1H, d, *J* = 8.6 Hz, H-2), 7.22 (1H, d, *J* = 8.6 Hz, H-3), 6.81 (1H, dd, *J* = 7.7, 1.1 Hz, H-6), 7.26 (1H, dd, *J* = 8.8, 7.7 Hz, H-7), 7.64 (1H, dd, *J* = 8.8, 1.1 Hz, H-8), 4.98 (1H, d, *J* = 7.8 Hz, H-1′), 3.53 (1H, dd, *J* = 9.0, 7.8 Hz, H-2′), 3.49 (1H, m, H-3′), 3.42 (1H, m, H-4′), 3.49 (1H, m, H-5′), 3.74 (1H, dd, *J* = 12.0, 5.8 Hz, H-6′), 3.94 (1H, dd, *J* = 12.0, 2.1 Hz, H-6′). ^13^C-NMR (CDCl_3_, 100 MHz) δ (ppm): 150.8 (C-1), 108.5 (C-2), 113.2 (C-3), 148.8 (C-4), 154.9 (C-5), 112.6 (C-6), 127.1 (C-7), 114.7 (C-8), 129.2 (C-9), 117.9 (C-10), 105.2 (C-1′), 75.2 (C-2′), 78.3 (C-3′), 71.6 (C-4′), 78.8 (C-5′), 62.8 (C-6′).

*1,4,8-Trihydroxy-3-naphthalenecarboxylic acid-1-O-β-d-glucopyranoside ethyl ester* (**18**). red powder, mp 136-138 °C;${\left[\alpha \right]}_{\text{D}}^{25}$ −12.3 (*c* 0.50, MeOH); UVλ_max_ = 243 nm; IR (KBr)ν_max_ 3401, 2956, 1646, 1506 cm^−1^; ^1^H-NMR and ^13^C-NMR data see Table 1; HR-ESI-MS (positive): *m*/*z* 433.1375 [M + Na]^+^ (calcd for C_19_H_22_NaO_10_, 433.1381).

*1,4,8-Trihydroxynaphthalene-1-O-β-d-[6'-O-(3'',4'',5''-trihydroxybenzoyl)] glucopyranoside* (**19**). Yellow amorphous powder, ^1^H-NMR (CD_3_OD, 400 MHz) δ (ppm): 6.50 (1H, d, *J* = 8.4 Hz, H-2), 7.18 (1H, d, *J* = 8.4 Hz, H-3), 7.68 (1H, dd, *J* = 8.0, 3.1 Hz, H-5), 7.23 (1H, dd, *J* = 8.0, 7.8 Hz, H-6), 6.80 (1H, dd, *J* = 8.2, 3.1 Hz, H-7), 4.96 (1H, d, *J* = 7.8 Hz, H-1′), 3.58 (1H, m, H-2′), 3.53 (1H, m, H-3′), 3.29 (1H, m, H-4′), 3.84 (1H, m, H-5′), 4.53 (1H, dd, *J* = 12.0, 6.9 Hz, H-6′a), 4.66 (1H, dd, *J* = 12.0, 2.1 Hz, H-6′b), 7.18 (2H, s, H-2″, H-6″). ^13^C-NMR (CDCl_3_, 100 MHz) δ (ppm): 148.8 (C-1), 108.6 (C-2), 113.3 (C-3), 150.7 (C-4), 114.7 (C-5), 127.1 (C-6), 112.2 (C-7), 154.8 (C-8), 117.9 (C-9), 128.9 (C-10), 105.4 (C-1′), 78.2 (C-2′), 75.1 (C-3′), 72.0 (C-4′), 76.2 (C-5′), 65.1 (C-6′), 168.6 (C-7″), 121.6 (C-1″), 110.4 (C-2″, C-6″), 146.7 (C-3″, C-5″), 140.3 (C-4″).

*(4S)-4-Hydroxy-α-tetralone-4-O-β-d-glucopyranoside* (**20**). White amorphous powder, ^1^H-NMR (CD_3_OD, 400 MHz) δ (ppm): 3.05 (1H, ddd, *J =* 17.8, 9.6, 4.8 Hz, H-2a), 2.62 (1H, ddd, *J =* 17.8, 6.8, 4.8 Hz, H-2b), 2.45 (1H, dddd, *J =* 13.2, 9.6, 4.8, 3.4 Hz, H-3a), 2.38 (1H, dddd, *J =* 13.2, 6.8, 6.2, 4.8 Hz, H-3b), 5.10 (1H, dd, *J =* 6.2, 3.4 Hz, H-4), 7.71 (1H, dd, *J =* 7.8, 1.6 Hz, H-5), 7.63 (1H, td, *J =* 7.8, 1.6 Hz, H-6), 7.47 (1H, td, *J =* 7.8, 1.6 Hz, H-7), 7.97 (1H, dd, *J =* 7.8, 1.6 Hz, H-8), 4.38 (1H, d, *J* = 7.8 Hz, H-1′), 3.24 (1H, dd, *J* = 8.5, 7.8 Hz, H-2′), 3.32 (1H, m, H-3′), 3.31 (1H, m, H-4′), 3.32 (1H, m, H-5′), 3.71 (1H, dd, *J* = 11.8, 5.3 Hz, H-6′a), 3.95 (1H, dd, *J* = 11.8, 1.0 Hz, H-6′b). ^13^C-NMR (CD_3_OD, 100 MHz) δ (ppm): 200.2 (C-1), 35.6 (C-2), 31.6 (C-3), 75.1 (C-4), 130.4 (C-5), 135.1 (C-6), 129.9 (C-7), 128.2 (C-8), 133.1 (C-9), 144.1 (C-10), 103.3 (C-1′), 75.4 (C-2′), 78.2 (C-3′), 71.9 (C-4′), 78.1 (C-5′), 63.0 (C-6′).

*(4S)-4,5-Dihydroxy-α-tetralone-4-O-β-d-glucopyranoside* (**21**). White amorphous powder, ^1^H-NMR (CD_3_OD, 400 MHz) δ (ppm): 3.12 (1H, ddd, *J =* 17.6, 14.0, 5.1 Hz, H-2a), 2.49 (1H, dt, *J =* 17.6, 3.3 Hz, H-2b), 2.19 (1H, tt, *J =* 14.0, 3.3 Hz, H-3a), 2.56 (1H, ddt, *J =* 14.0, 5.1, 3.3 Hz, H-3b), 5.41 (1H, t, *J* = 3.3 Hz, H-4), 7.12 (1H, dd, *J =* 7.9, 1.1 Hz, H-6), 7.30 (1H, t, *J =* 7.9 Hz, H-7), 7.48 (1H, dd, *J =* 7.9, 1.1 Hz, H-8), 4.62 (1H, d, *J* = 7.8 Hz, H-1′), 3.19 (1H, dd, *J* = 8.7, 7.8 Hz, H-2′), 3.35 (1H, m, H-3′), 3.36 (1H, m, H-4′), 3.36 (1H, m, H-5′), 3.75 (1H, dd, *J* = 12.2, 4.8 Hz, H-6′a), 3.90 (1H, dd, *J* = 12.2, 1.8 Hz, H-6′b). ^13^C-NMR (CD_3_OD, 100 MHz) δ (ppm): 201.2 (C-1), 34.0 (C-2), 30.3 (C-3), 70.1 (C-4), 156.8 (C-5), 122.3 (C-6), 130.7 (C-7), 119.2 (C-8), 134.7 (C-9), 130.0 (C-10), 104.1 (C-1′), 75. 6 (C-2′), 78.1 (C-3′), 71.6 (C-4′), 78.1 (C-5′), 62.9 (C-6′).

*(4S)-4,6-Dihydroxy-α-tetralone-4-O-β-d-glucopyranoside* (**22**). white amorphous powder, ^1^H-NMR (CD_3_OD, 400 MHz) δ (ppm): 2.97 (1H, ddd, *J =* 17.7, 9.7, 5.1 Hz, H-2a), 2.52 (1H, ddd, *J =* 17.7, 6.4, 4.8 Hz, H-2b), 2.38 (1H, dddd, *J =* 13.6, 9.6, 4.8, 3.5 Hz, H-3a), 2.30 (1H, dddd, *J =* 13.6, 6.4, 6.2, 5.1 Hz, H-3b), 5.02 (1H, dd, *J* = 6.2, 3.5 Hz, H-4), 7.04 (1H, d, *J* = 2.2 Hz, H-5), 6.85 (1H, dd, *J* = 8.4, 2.2 Hz, H-7), 7.88 (1H, d, *J* = 8.4 Hz, H-8), 4.38 (1H, d, *J* = 7.8 Hz, H-1′), 3.26 (1H, dd, *J* = 8.7, 7.8 Hz, H-2′), 3.33 (1H, t, *J* = 8.7 Hz, H-3′), 3.30 (1H, m, H-4′), 3.28 (1H, m, H-5′), 3.70 (1H, dd, *J* = 11.9, 5.8 Hz, H-6′a), 3.93 (1H, dd, *J* = 11.9, 1.6 Hz, H-6′b). ^13^C-NMR (CD_3_OD, 100 MHz) δ (ppm): 199.6 (C-1), 35.3 (C-2), 31.5 (C-3), 75.1 (C-4), 116.0 (C-5), 164.4 (C-6), 117.0 (C-7), 131.1 (C-8), 125.5 (C-9), 146.4 (C-10), 103.0 (C-1′), 75.3 (C-2′), 78.2 (C-3′), 71.9 (C-4′), 78.1 (C-5′), 63.2 (C-6′).

*(4S)-4,5,8-Trihydroxy-α-tetralone-4-O-β-d-glucopyranoside* (**23**). White amorphous powder, ^1^H-NMR (CD_3_OD, 400 MHz) δ (ppm): 3.22 (1H, ddd, *J =* 18.0, 13.8, 4.8 Hz, H-2a), 2.52 (1H, dt, *J =* 18.0, 3.8 Hz, H-2b), 2.17 (1H, tdd, *J =* 13.8, 3.8, 3.0 Hz, H-3a), 2.48 (1H, m, H-3b), 5.40 (1H, t, *J* = 3.0 Hz, H-4), 7.11 (1H, d, *J* = 9.1 Hz, H-6), 6.81 (1H, d, *J* = 9.1 Hz, H-7), 4.52 (1H, d, *J* = 7.8 Hz, H-1′), 3.17 (1H, dd, *J* = 8.9, 7.8 Hz, H-2′), 3.32 (1H, m, H-3′), 3.32 (1H, m, H-4′), 3.32 (1H, m, H-5′), 3.72 (1H, dd, *J* = 12.1, 4.8 Hz, H-6′a), 3.91 (1H, dd, *J* = 12.1, 1.8 Hz, H-6′b). ^13^C-NMR (CD_3_OD, 100 MHz) δ (ppm): 207.2 (C-1), 34.3 (C-2), 29.9 (C-3), 69.3 (C-4), 148.6 (C-5), 127.0 (C-6), 119.6 (C-7), 157.3 (C-8), 116.9 (C-9), 127.5 (C-10), 103.4 (C-1′), 75.4 (C-2′), 78.2 (C-3′), 71.7 (C-4′), 78.1 (C-5′), 62.8 (C-6′).

*(4S)-4,5,8-Trihydroxy-α-tetralone-5-O-β-d-[6'-O-(3",4",5"-trihydroxybenzoyl)] glucopyranoside* (**24**). Light yellow amorphous powder, ^1^H-NMR (CD_3_OD, 400 MHz) δ (ppm): 2.49 (1H, dd, *J =* 17.6, 3.5 Hz, H-2a), 3.03 (1H, ddd, *J =* 17.6, 11.8, 6.5 Hz, H-2b), 2.15 (1H, m, H-3), 5.34 (1H, t, *J* = 3.2 Hz, H-4), 7.42 (1H, d, *J* = 9.2 Hz, H-6), 6.76 (1H, d, *J* = 9.2 Hz, H-7), 4.78 (1H, d, *J* = 7.7 Hz, H-1′), 3.55 (1H, t, *J* = 8.0 Hz, H-2′), 3.51 (1H, m, H-3′), 3.45 (1H, m, H-4′), 3.68 (1H, td, *J* = 7.8, 1.9 Hz, H-5′), 4.45 (1H, dd, *J* = 11.8, 6.8 Hz, H-6′a), 4.55 (1H, dd, *J* = 11.8, 2.1 Hz, H-6′b), 7.07 (2H, s, H-2″, H-6″). ^13^C-NMR (CD_3_OD, 100 MHz) δ (ppm): 206.2 (C-1), 33.5 (C-2), 30.3 (C-3), 61.1 (C-4), 148.6 (C-5), 129.1 (C-6), 119.4 (C-7), 159.5 (C-8), 116.1 (C-9), 135.1 (C-10), 104.5 (C-1′), 75.3 (C-2′), 77.9 (C-3′), 71.7 (C-4′), 75.8 (C-5′), 64.6 (C-6′), 121.0 (C-1″), 110.4 (C-2″), 146.9 (C-3″), 140.4 (C-4″), 146.9 (C-5″), 110.3 (C-6″), 168.3 (C-7″).

*(4S)-4-Hydroxy-α-tetralone-4-O-β-d-(6'-O-4''-hydroxybenzoyl) glucopyranoside* (**25**). yellow powder, mp 128–132 °C; ${\left[\alpha \right]}_{\text{D}}^{25}$ −28.2 (*c* 0.50, MeOH); UVλ_max_ = 262 nm; CD (MeOH) λ_max_ (∆ε): 236 nm (−12.6), 258 nm (+1.23). IR (KBr)ν_max_ 3396, 1728, 1260, 1180 cm^−1^; ^1^H-NMR and ^13^C-NMR data see Table 1; HR-ESI-MS (positive): *m*/*z* 445.0262 [M + H]^+^ (calcd for C_23_H_25_O_9_, 445.0267).

*(4S)-4,5-Dihydroxy-α-tetralone-4-O-β-d-(6'-O-4''-hydroxybenzoyl) glucopyranoside* (**26**). yellow powder, mp 132–135 °C; ${\left[\alpha \right]}_{\text{D}}^{25}$ −30.2 (*c* 0.52, MeOH); UVλ_max_ = 274 nm; CD (MeOH) λ_max_ (∆ε): 234 nm (−8.60), 261 nm (+2.21). IR (KBr)ν_max_ 3420, 2960, 1726, 1255, 1135 cm^−1^; ^1^H-NMR and ^13^C-NMR data see Table 1; HR-ESI-MS (positive): *m*/*z* 483.4276 [M + Na]^+^ (calcd for C_23_H_24_NaO_10_, 483.4279).

*(4S)-4,5,8-Thihydroxy-α-tetralone-5-O-β-d-(6'-O-4''-hydroxybenzoyl) glucopyranoside* (**27**). yellow powder, mp 145–148 °C; ${\left[\alpha \right]}_{\text{D}}^{25}$ −31.4 (*c* 0.47, MeOH); UVλ_max_ = 264 nm; CD (MeOH) λ_max_ (∆ε): 241 nm (−9.46), 266 nm (+0.93), 271 nm (−0.55), 292 nm (+0.35). IR (KBr)ν_max_ 3400, 2994, 1725, 1056 cm^−1^; HR-ESI-MS (positive): *m*/*z* 477.1058 [M + H]^+^ (calcd for C_23_H_25_O_11_, 477.1054).

### 3.5. Acid Hydrolysis and Sugar Analysis

Compounds **18**, **25**–**27** (1.5 mg) were refluxed with 1.0 mol/L HCl (5 mL, dioxane/H_2_O, *v*/*v*) for 7 h. After filtration, the acid aqueous layer was neutralized with 5% NaOH and desalted with Sephadex LH-20 to obtain the sugar residue (0.8 mg). The residues were dissolved in pyridine (5 mL) and 1-(trimetrylsilyl)-imidazole (0.5 mL) at 60 °C for 10 min. The reaction mixtures were dried with a stream of N_2_, the residues were partitioned between CHCl_2_ and H_2_O. The organic layers were analyzed by GC using an L-Chirasil-Val column (0.32 mm × 25 m). Temperatures of injector and detector were maintained at 200 °C. A temperature gradient system was used for the oven; the initial temperature remained at 100 °C for 1 min and then was raised to 180 °C at the rate of 5 °C/min. Peaks of the hydrolysate of **18** and **25**–**27** were respectively detected at 14.72 min, 14.72 min, 14.73 min, 14.72 min. The final result was to compare the retention time of authentic sample of d-glucose (SigmaAldrich, St. Louis, MO, USA) treated in the same manner with 1-(trimetrylsilyl)-imidazole in pyridine which was detected at 14.72 min. Thus, it was concluded that all the sugar moieties of **18** and **25**–**27** are d-glucose.

### 3.6. Cytotoxicity Assays

#### 3.6.1. Cell Culture

The cytotoxicity of compounds **1**–**27** was performed against human liver carcinoma cells (HepG-2) by MTT assay [25,26]. HepG-2 cell line was maintained in DMEM supplemented with 10% fetal bovine serum (FBS), 100 units/mL penicillin, and 100 μg/mL streptomycin (Gibco-BRL). The cells were incubated in 5% CO_2_ humidified at 37 °C for growth.

#### 3.6.2. Measurement of Cell Proliferation by MTT Assay

HepG-2 cells in logarithmic growth phase were seeded in a 96-well microtiter plates and kept overnight for attachment. Twenty-seven compounds and positive control (cisplatin), dissolved in dimethyl sulfoxide, were diluted to various concentrations with Dulbecco’s Modified Eagle Medium (DMEM) from 200 to 0.5 μM for 24 h. The optical density (OD) was measured at 570 nm using a multiscan microplate reader. All experiments were performed in triplicate. Data were expressed as the concentration required for inhibiting growth of HepG-2 by 50% (IC_50_).

## 4. Conclusions

Twenty-seven naphthoquinones and their derivatives, including four new naphthalenyl glucosides and twenty-three known compounds, have been isolated with the aim of exploring the relationship between cytotoxicity and structures. The results indicated that in naphthoquinones with 2,3-unsaturated moieties, the position of the substituents was at the aryl ring portion or the quinone ring portion of naphthoquinone played an important role in the cytotoxic activity. Moreover, the type of substituents also had an effect on the activity. And in general, when these compounds were substituted with the phenolic hydroxyl group, they had stronger activity against the HepG-2 cells. Napthoquinone glycosides had no activity or weaker activity. So far, we are not able to definitely confirm that the type of saccharide is an essential factor for cytotoxic activity, since compounds obtained are all substituted with glucose. These results will provide experimental bases for further structural modifications to yield better active derivatives.
